# Enhanced Photothermal Based-Heat Retention in Regenerated Cellulose Fibers via Ceramic Particles and Polyelectrolyte Binders-Based Surface Functionalization

**DOI:** 10.3390/polym17070961

**Published:** 2025-04-01

**Authors:** Özkan Yapar, Ajra Hadela, Alenka Ojstršek, Aleksandra Lobnik

**Affiliations:** 1 Faculty of Mechanical Engineering, University of Maribor, Smetanova Ulica 17, 2000 Maribor, Slovenia; 2 Institute for Environmental Protection and Sensors (IOS) Ltd., Beloruska Ulica 7, 2000 Maribor, Slovenia

**Keywords:** regenerated cellulose fibers, RCFs, ceramic particles, zirconium carbide, ZrC, surface functionalization, heat generation and retention

## Abstract

There has been growing interest and increasing attention in the field of functional clothing textiles, particularly in product and process development, as well as innovations in heat-generating, retaining, and releasing fibers to maintain a healthy body temperature without relying on unsustainable energy sources. This study, for the first time, reports the various physio-mechanical properties of surface-functionalized regenerated cellulose fibers (RCFs) coated with ceramic particles. The coating imparts photothermal conversion-based heat generation and retention properties with the aid of polyelectrolyte binders. In this design, ZrC enables the conversion of light energy into thermal energy, providing heat for the human body. A feasible coating process was employed, utilizing industrially feasible exhaustion methods to deposit the ZrC particles onto the RCF surface in conjunction with two distinctive polymeric binders, specifically polyethyleneimine (PEI) and polydiallyldimethylammonium chloride (polyDADMAC). The morphological characteristics and tensile properties of the coated RCFs were analyzed via scanning electron microscopy (SEM) and single-fiber tensile testing. Heat retention and release behaviors of a bundle of fiber samples were assessed using infrared (IR) imaging and an IR emission lamp setup. The SEM results confirmed the successful coating of the ZrC particles on the surface of the RCF samples, influencing negligible on their physical–mechanical properties. The heat retention of the coated RCFs with ZrC and both binders was higher than that of reference regenerated cellulose fibers (RCFs), demonstrating their effective heat generation, retention, and heat release properties. Based on the highlighted prominent results for the coated RCFs, these findings highlight the suitability of the developed functional clothing textiles for targeted applications in non-extreme thermal conditions, ensuring thermo-physiological comfort by maintaining body temperature within a tolerable thermal range (36.5–37.5 °C), in contrast to studies reporting significantly higher temperatures (50–78 °C) for extreme thermal conditions.

## 1. Introduction

Advanced technology and rising living standards have driven growing interest in functional clothing textiles across various fields, including sports, healthcare, personal protection, and wearable technology [1,2,3,4]. Photothermal conversion has emerged as a promising technology for these functional textiles, leveraging solar energy as a clean, abundant, and renewable source. As solar energy can be converted into heat, integrating this mechanism into that kind of apparel textiles can enhance thermal management, enabling garments to provide continuous warmth, especially in cold conditions [5,6,7,8]. The integration of photothermal materials into functional clothing textiles enables the conversion of solar light into heat, enhancing the thermal management properties of fabrics. Over the past decade, the development of photothermal conversion composites on textile surfaces, particularly using inorganic ceramic materials, has gained significant attention for applications in smart textiles, purification, therapy, and heat preservation [5,9,10,11].

For targeting photothermal-based heat retention with or without release properties, previous studies have reported the use of various materials, including bio-ceramic additives [12], graphene oxide (GO) [13], aluminum oxide (Al_2_O_3_)/graphite [14,15], polyvinyl butyral/zirconium carbide/alumina oxide (PVB/ZrC) [16], cesium–tungsten bronze (Cs*_x_*WO_3_) nanoparticles [8], copper sulfide (CuS) [17], aluminum oxide (Al_2_O_3_)/antimony-doped tin oxide (ATO)/titanium dioxide (TiO_2_) [18], and reduced graphene oxide/bismuth sulfide (rGO/Bi_2_S_3_) nanocomposite particles [19], among others.

Among the previously presented materials, zirconium carbide (ZrC) is interesting and a known ceramic material with a heat capacity of 37.442 J/mol·°C [20] and the properties of thermal conductivity of 17.5–31.5 W·m^−1^·°C^−1^ [21]. Metallic ZrC catalysts have nearly no bandgap, a high carrier density, and exhibit broad-spectrum absorption. They also demonstrate excellent thermo-chemical stability, with efficient light absorption and solar energy conversion across the visible and near-infrared spectra [22,23,24,25].

In this context, utilizing ZrC particles can be categorized as the most suitable for applications in both (semi-)extreme and non-extreme thermal conditions. In the context of extreme conditions, several papers [5,7,26,27] have reported materials intended for (multifunctional) clothing textiles, including applications in sports and winter clothing [5], a broad range of uses including clothing [26], multifunctional textiles [27], and clothing textiles designed for cold climates [7]. For non-extreme thermal conditions, only a few papers have been published intended to apply their reported materials for clothing textiles ZrC particles [28,29,30] and they are included in Table 1. All these mentioned papers are difficult to compare due to their varying test setups, different protocols, and distinct or partially overlapping experimental conditions, such as IR lamp or camera distance and the use of thermo-sensors, for assessing photothermal-based heat retention properties. However, we partially compared our coated RCFs with reported viscose fibers from a Japanese company’s datasheet, as their test setup, particularly the IR lamp distance (50 cm), IR camera distance (20 cm), and heating test duration (20 min), was the closest to ours. A rare study was published focusing on the influence of test setup parameters on heat retention and release property assessments, particularly their impact on the maximum surface temperature of samples. Kim et al. [31] reported a study on ZrC/Al_2_O_3_ + graphite-embedded heat storage woven fabrics made from ZrC- and Al_2_O_3_-embedded heat storage PET filaments [31]. In previous studies, ZrC particles were incorporated only into polyethylene terephthalate (PET) [29] and polyester (PES) textiles [28], with limited research exploring their coating on cellulosic materials [30] for heat generation, retention, and heat release properties. This highlights the critical role of IR heating distance in influencing results, making direct comparisons with other studies challenging. Therefore, our study was only compared with the reported dataset from a Japanese company due to the same IR heating distance and similar test setup (Table 1).

In reported studies on the deposition of materials onto cellulosic substrates for heat generation, retention, and heat release, Zhang et al. [3] investigated the adhesion of 4% zirconium carbide (ZrC) to cotton yarn using a polyurethane (PU) sizing coating method.

Here, we report for the first time on the surface functionalization of regenerated cellulose fibers (RCFs), using polyelectrolyte-based polymeric binders like PEI and polyDADMAC. This study evaluates their photothermal conversion capabilities, including heat generation, retention, and release properties. Specifically, the synergistic effects of pre-coating RCF samples with a calcium chloride (CaCl_2_) and carboxymethyl cellulose (CMC) mixture on the deposition of ZrC via polyDADMAC have not been previously reported. This makes the study the first of its kind and a novel contribution to the field.

The ultimate surface temperature values of 30% *w*/*v* ZrC-coated RCFs (rinsed) with a 1% *w*/*v* polyDADMAC binder (pre-coating of the RCFs with CaCl_2_ and CMC mixture) exhibited a maximum surface temperature of 39.08 °C, demonstrating a heat increase of 6.87 °C (+21.34%). Similarly, the 30% *w*/*v* ZrC-coated RCFs (rinsed) with a 1% *w*/*v* PEI binder reached 37.07 °C, compared to 32.21 °C for the reference RC fiber, demonstrating a heat increase of 4.86 °C (+15.09%) under 20 min IR irradiation exposure from the IR lamp. Additionally, our RCFs coated with PEI and polyDADMAC binders resulted in an approximate 1 °C increase in irradiated surface temperature compared to reference RCF samples under identical IR exposure conditions. This suggests that these polyelectrolyte-based polymeric binders alone do not have a substantial impact on the heat release and storage properties of ZrC-coated RCFs, though their minor enhancement effect can still be noted.

Moreover, when the ZrC particles were applied to the RCFs without polyelectrolyte binders by mixing ZrC with deionized (DI) water in a glass beaker at room temperature for at least 12 h with magnetic stirring, SEM micrographs revealed minimal attachment of ZrC onto RC fiber surfaces, especially after their rinsing. Therefore, our study does not report specific results for ZrC coating without polyelectrolyte binders, as ZrC alone was the least efficient for surface attachment onto RCFs. Consequently, PEI and polyDADMAC binders were employed to facilitate ZrC coating. Additionally, SEM images revealed that 1% (*w*/*v*) polyDADMAC with 10% (*w*/*v*) ZrC was less effective in securing ZrC attachment on RCF surfaces compared to 1% (*w*/*v*) PEI with 10% (*w*/*v*) ZrC. Thus, to improve the cross-linking efficiency of polyDADMAC and enhance ZrC deposition, RCFs were pre-coated with a CaCl_2_ and CMC mixture before applying 1% (*w*/*v*) polyDADMAC.

Furthermore, the highest recorded values of our coated RCF samples (rinsed), 39.08 °C and 37.07 °C, are comparable to the data published by Daiwabo Rayon Co., Ltd., Osaka, Japan (excluding factors such as yarn spinnability and nonwoven producibility) in their datasheet, which reported heat-generating and heat-retaining fibers tested under similar conditions, as shown in Table 1. Similar to our study, they also used an IR lamp positioned 50 cm from the sample with a 20 min light-on duration and reported that the maximum surface temperature of their fibers reached approximately 38 °C, compared to 33 °C for their reference RC fiber (viscose), demonstrating a heat increase of approximately 5 °C (+15.15%). Notably, our polyDADMAC-coated RCFs (rinsed) with 30% *w*/*v* ZrC (pre-coated with the CaCl_2_ and CMC mixture) exhibited approximately 1 °C higher heat retention than the commercially available fiber (i.e., 39.08 °C). The commercial fiber was tested in spunlace nonwoven form. Since the type of produced test specimens (e.g., nonwoven, woven, knitted) significantly influences heat generation and retention properties, our results can be considered even more favorable than those of commercial RC (viscose) fibers from the reported company, as our tests were conducted on fiber bundles rather than denser nonwoven structures. Generally, denser, and more compact fiber structures exhibit higher heat generation and retention values due to their larger IR-illuminated area and increased surface temperature [26].

Notably, the ultimate surface temperature values of our coated RCF samples are promising for further product development, as our heat retention test results met their targeted values. Our study specifically aimed to develop functional regenerated cellulose fibers (RCFs) that help maintain body temperature within a tolerable range (36.5–37.5 °C) to provide thermo-physiological comfort [32] for non-extreme thermal conditions, in contrast to previously reported studies that focused on significantly higher temperatures (50–78 °C) [5,7,26,27], which are more suitable for extreme or semi-extreme thermal conditions.

As part of our research, the morphological characteristics, and tensile properties of the RCF samples were analyzed using scanning electron microscopy (SEM) and single-fiber tensile testing. Photothermal-based heat retention and release behaviors were assessed via infrared (IR) imaging and a heat emission lamp setup. Notably, our sample testing duration was 40 min, including 20 min of heating and 20 min of cooling which is the longest reported in similar studies. This extended testing duration allowed for a broader evaluation of ZrC-coated RCFs’ performance. SEM results confirmed the successful deposition of ZrC particles on the surface of coated RCFs, with minimal impact on their physical–mechanical properties. Additionally, the surface coating of regenerated cellulose fibers, particularly those using polyethyleneimine (PEI) binders (with or without ZrC), was further analyzed via X-ray photoelectron spectroscopy (XPS) to provide complementary data on the PEI-coated RCF samples.

## 2. Experimental

### 2.1. Materials

Regenerated cellulose fibers (RCFs) (CV, bright type, Europe) with a fineness of 0.9–17 dtex and a cut length of 38–150 mm were purchased from Lenzing AG (Lenzing, Austria). Zirconium carbide (ZrC) particles, synthesized by Alfa Aesar (Karlsruhe, Germany), were obtained as an ultrafine powder (99.5% purity, Hf < 200 ppm). To coat the RCFs with ZrC particles, two polymeric binders were selected: polyethyleneimine (PEI, Mw 25,000 by light scattering) and polydiallyldimethylammonium chloride (polyDADMAC, 20 wt.% in H_2_O, Mw 200,000–350,000), both purchased from Sigma-Aldrich (Vienna, Austria).

To enhance the coatability of the polyDADMAC solution on RCF surfaces with ZrC, sodium carboxymethyl cellulose (Na-CMC, average Mw ~90,000) and calcium chloride (CaCl_2_), both purchased from Sigma-Aldrich (Vienna, Austria), were used for pre-coating the RCFs. Additionally, a textile auxiliary agent, Lavotan DSU, was obtained from Bezema AG (Montlingen, Switzerland) for rinsing the RCF samples. All chemicals were used as received, without further purification.

### 2.2. Surface Coating of RCFs

Before performing the coating process, aqueous stock solutions of polyethyleneimine (PEI, 1.0% *w*/*v*) and polyDADMAC (1.0% *w*/*v*) were prepared by vigorously mixing with deionized (DI) water in a glass beaker at room temperature for at least 12 h using a magnetic stirrer.

RCFs were coated using a one-bath exhaustion process in a sealed, stainless-steel dye pot (250 cm³ capacity) housed in a Labomat (W. Mathis AG, Oberhasli, Switzerland) laboratory-scale apparatus with 1200 rpm bath circulation. The initial bath contained 0.1% or 0.5% owf (on the weight of fibers) of ZrC, 9% owf of an individual binder (PEI or polyDADMAC), and 1 g/L of a pH regulator (Sandacid DSB, Clariant, Muttenz, Switzerland) to adjust the pH to 4.5–5.0. The coating procedure was conducted at 65 °C (with a heating rate of 3 °C/min) for 60 min, using a liquor-to-fiber weight ratio of 20:1 (190 mL of DI water per 9.5 g of fibers). The pre-coating of RCFs with 0.5 M CaCl_2_ + 5% *w*/*v* CMC mixtures followed the same protocol and processing conditions.

**Figure 1:**
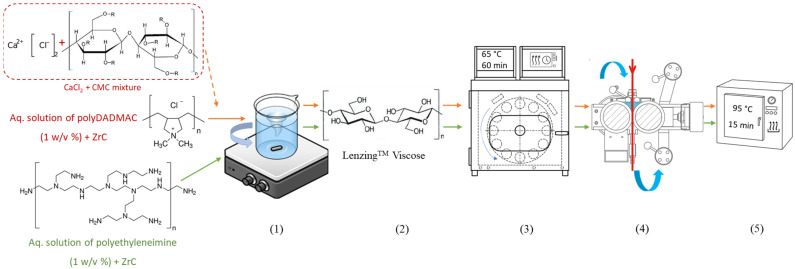
Schematic representation of the RCF coating process using two distinctive polymeric binders and ZrC particle adsorption. (1) Preparation of the coating medium in a glass beaker with a magnetic stirrer at room temperature. (2) Immersion of RCF bundles into the prepared solution. (3) Coating using the Labomat device. (4) Squeezing and padding of wet RCF samples between rotating rollers at ambient temperature. (5) Drying of coated samples in a laboratory oven.

At the end of the treatment, the coated fibers were removed from the bath, pressed between two rollers of a padding device (Mathis AG, Oberhasli, Switzerland) to eliminate excess water, and air-dried at room temperature. To remove any unbound reactants, the treated samples were washed at 50 °C for 5 min (bath-to-fiber weight ratio of 20:1) using 2 g/L of a non-ionic wetting agent, rinsed with DI water, oven-dried at 95 °C for 15 min, and finally air-dried at room temperature for further analysis. A schematic illustration of the coating process is presented in Figure 1. Abbreviations and descriptions of uncoated, pre-coated, and coated RCF samples fabricated in the experiments are listed in Table 2.

## 3. Characterization Methods

### 3.1. Field-Emission Scanning Electron Microscopy

The morphology of the reference RCF (CV) and coated RCFs were examined using field-emission scanning electron microscopy (FE-SEM, Carl Zeiss SUPRA 35 VP, Oberkochen, Germany) equipped with a secondary electron detector at an accelerating voltage of 5 kV. Images were recorded at a working distance of 4.5 mm. The fiber samples were attached to sample holders using double-sided carbon adhesive tape, and no sputtering was performed on the sample surfaces for imaging.

### 3.2. Tensile Properties of RCFs

To evaluate the influence of coated ZrC particles and polymer binders on the mechanical performance of reference RCFs, tensile properties were assessed. Prior to testing, all fiber samples were conditioned for at least 48 h in a standard atmosphere within a climatic chamber, following ISO/R 139 [33] at 20 ± 2 °C and 65 ± 2% relative humidity (RH).

The linear density (titer) of individual fiber samples was determined using a Vibroskop 400 (Lenzing Instruments, Gampern, Austria) in accordance with ISO 1973:2021 [34]. Tensile properties were measured with an electronic dynamometer, Vibrodyn 400 (Lenzing Instruments, Gampern, Austria), following SIST ISO 5079: 2020 [35]. Each sample batch was tested 25 times, and the average value was reported.

The impact of the coating procedures on the mechanical properties of the RCFs was assessed by measuring force and elongation using a gauge length of 20 mm, a pre-tension weight of 150 mg, and a testing speed of 20 mm/min.

### 3.3. Photothermal Property Measurements of RCFs

Photothermal conversion-based heat retention and release measurements under light irradiation were performed using an infrared heat lamp (Philips, 100 W 230 V R95 IR, Amsterdam, The Netherlands) and an infrared (IR) camera (Optris PI400, Optris GmbH & Co. KG, Berlin, Germany). The IR lamp simulated sunlight exposure on the fiber samples, as IR radiation contributes to thermal effects and accounts for approximately 53% of solar energy [7]. Surface temperatures of the fiber samples were recorded using the IR camera [7].

**Figure 2:**
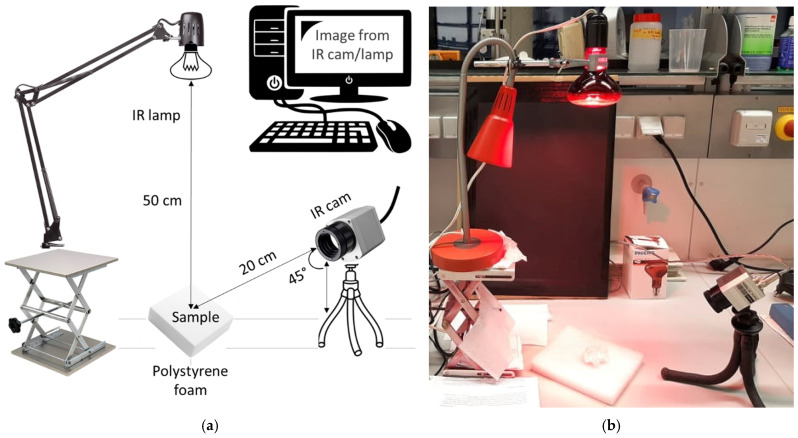
Schematic diagram (**a**) and the measuring apparatus (**b**) of the testing setup.

Figure 2 presents a schematic representation of the experimental setup for capturing thermal images of the fiber sample batches. To ensure consistency and accuracy in photo-induced heat generation experiments, fiber samples were prepared as bundles (1 g, dry weight), placed on a heat-insulating polystyrene foam plate, and tested under controlled laboratory conditions (20 ± 2 °C, RH 50% ± 2%). The IR camera was positioned at a 45° angle towards the fiber bundle, maintaining a distance of 20 cm, as depicted in Figure 2. The IR lamp was fixed vertically above the specimen at a height of 50 cm. Each test lasted 40 min, comprising 20 min of IR exposure followed by 20 min with the lamp switched off. Temperature variations on the fiber surfaces were continuously monitored and manually recorded at 10 s intervals using the IR camera.

The measurement points for each fiber sample batch were selected at the center of the specimens, with a specific circled area chosen to ensure homogeneous data collection. For each IR camera measurement, the mean surface temperature of each specimen was determined using the camera’s software (Optris PIX Connect, Optris GmbH & Co. KG, Berlin, Germany) and manually recorded, reducing localized hot spots and ensuring consistency. This approach enabled the calculation of mean temperature values based on IR emissions detected by the IR camera. Ideally, the experiments would have been conducted in absolute darkness to eliminate ambient temperature influences, such as laboratory lighting, which can affect IR measurements. However, since such a setup was unavailable, all samples were tested under identical laboratory conditions to ensure reliable comparisons across datasets.

The temperature difference was calculated using the following equation:

Δ*T* = (*T*_10 min_ − *T*_0 min_) coated − (*T*_10 min_ − *T*_0 min_) uncoated

### 3.4. X-Ray Photoelectron Spectroscopy (XPS) Analysis

The surface chemical analysis of selected samples, RCFs, and PEI-coated fibers with and without ZrC particles, was performed using an XPS instrument (Kratos Supra+, Shimadzu Corporation, Kyoto, Japan). The base pressure in the XPS analysis chamber was maintained at approximately 2 × 10^−9^ torr. Each sample batch was excited with monochromatic Al Kα radiation over a large acquisition area of 300 × 700 μm. Photoelectrons were detected using a hemispherical analyzer positioned at a 90° take-off angle relative to the sample surface, with an estimated detection depth of a few nanometers.

Survey and high-resolution spectra were recorded at pass energies of 160 eV and 20 eV, respectively. All samples were mounted on Cu tape, and an additional electron gun was employed for surface charge neutralization during measurements to counteract charging effects in non-conductive samples. The binding energy scale was corrected by referencing the C–C/C–H peak in the C1s spectrum at 284.8 eV. Quantitative analysis was performed using the Shirley background correction, with elemental concentrations derived from the survey scan spectra, averaging five sweeps per spectrum. Each sample was measured three times to ensure repeatability.

## 4. Results and Discussions

### 4.1. Surface and Morphological Characterization of RCFs

**Figure 3:**
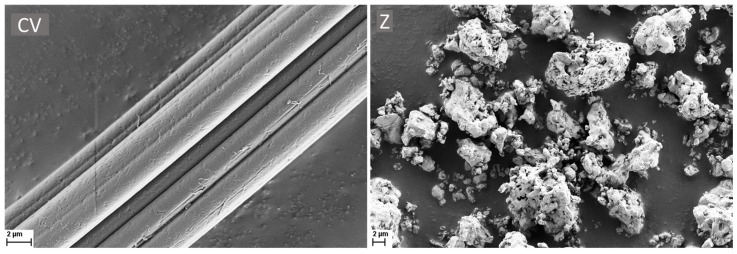
SEM micrographs of the reference RCF (CV) on the **left** and ZrC (Z) particles on the **right**, captured with a scale bar of 2 µm.

The surface morphologies of the RCFs and ZrC particles were examined using SEM micrographs, as shown in Figure 3. The RCF surface exhibited grooves, whereas the ZrC particles displayed micro-sized structures of varying shapes and sizes.

All coated RCF samples with ZrC particles (a_1_, a_1.1_, a_2_, a_2.1_, b_1_, b_1.1_, bx_1_, bx_1.1_) exhibited visible coatings on the fiber surfaces, confirming successful deposition without noticeable damage to the reference fibers. Additionally, ZrC particle agglomeration was observed across all these coated RCF samples (a_1_, a_1.1_, a_2_, a_2.1_, b_1_, b_1.1_, bx_1_, bx_1.1_), likely due to variations in particle size distribution. Similar agglomeration was reported by Wang et al. [27], where polyurethane/zirconium carbide (PU/ZrC) coatings were applied to cotton ply yarn via a sizing method for IR photothermal conversion. Their SEM images showed PU-coated yarns with ZrC particles forming fine layers with localized agglomerations [27].

Overall, the rinsing steps had a noticeable effect on the samples (a_1.1_, a_2.1_, b_1.1_, bx_1.1_), likely due to interactions between the hydroxyl groups of the PEI and polyDADMAC binding polymers. However, ZrC particles remained visible on those coated RCF samples after rinsing. Additionally, polyDADMAC-coated RCF fiber surfaces (b_1_, b_1.1_) exhibited lower particle retention compared to samples pre-coated with 0.5 M CaCl_2_ + 5% *w*/*v* CMC mixtures (bx_1_, bx_1.1_), indicating a higher degree of particle loss. This suggests a slight cross-linking effect of the CaCl_2_ + CMC mixtures with polyDADMAC, enhancing ZrC particle adsorption and retention on the surface of our RCF samples.

**Figure 4:**
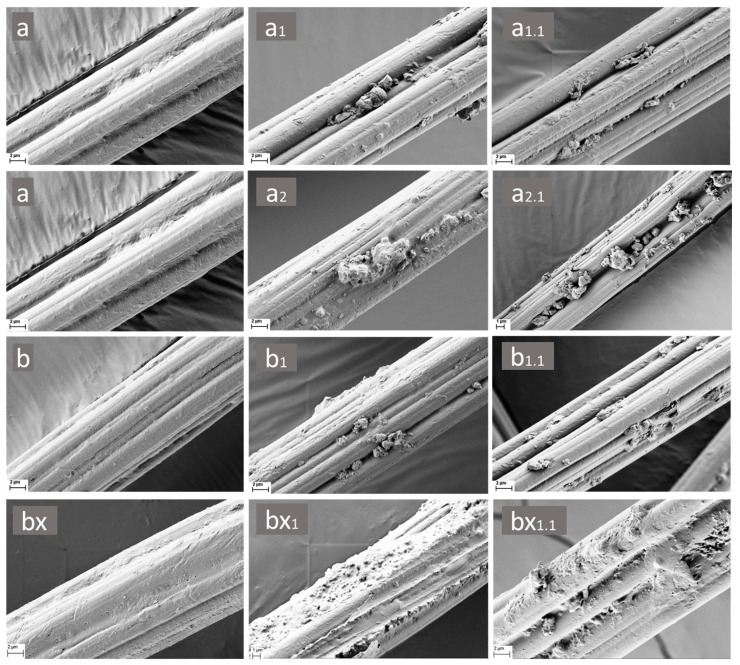
SEM micrographs of coated fibers prepared using aqueous PEI (**a**,**a_1_**,**a_1.1_**), polyDADMAC (**b**,**b_1_**,**b_1.1_**), and a CMC and CaCl_2_ mixture-assisted polyDADMAC coating (**bx**,**bx_1_**,**bx_1.1_**), with and without ZrC particles. Images (**a**,**b**,**bx**) depict the fiber surfaces without ZrC particles, while (**a_1_**,**a_2_**,**b_1_**,**bx_1_**) correspond to coated RCF samples before rinsing. The images (**a_1.1_**,**a_2.1_**,**b_1.1_**,**bx_1.1_**) represent ZrC-coated fiber samples after the rinsing step.

To support the findings for PEI-coated RCFs (a) with 30% *w*/*v* ZrC particles (a_2_) based on their SEM micrograph analysis (Figure 4), XPS analysis was conducted. As a selective and sensitive surface characterization technique, XPS was utilized to determine the surface composition of the PEI-coated RCF samples and to investigate the embedding of ZrC particles. 

RCF samples coated with 1% *w*/*v* PEI solution (a, a_1_, a_1.1_, a_2_, a_2.1_) exhibited well-covered fiber surfaces, particularly with the inclusion of 30% *w*/*v* ZrC particles. Notably, RCFs pre-coated with 0.5 M CaCl_2_ + 5% *w*/*v* CMC mixtures before applying the 1% *w*/*v* polyDADMAC solution (bx, bx_1_, bx_1.1_) showed a higher visual coating level compared to fibers coated solely with polyDADMAC (b, b_1_, b_1.1_), as indicated by the filled grooves of the reference RCFs (CV). 

**Figure 5:**
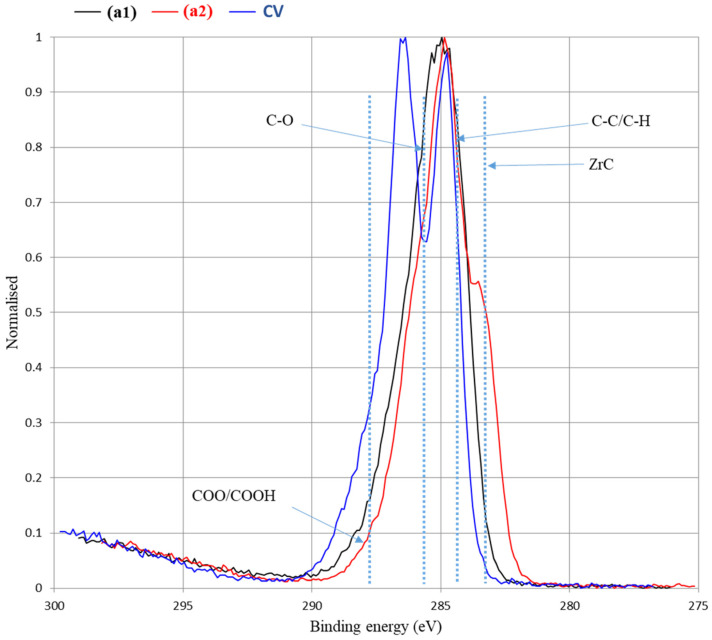
High-resolution XPS spectra of reference RCFs (CV), PEI-coated RCFs (a_1_), and PEI-coated RCFs with 30% *w*/*v* ZrC (a_2_).

The elemental surface compositions of the reference RCFs and the PEI-coated RCFs are shown in Table 3. Survey spectra, presented in Figure 5, were measured to determine the elemental composition. For all PEI-coated RCFs, with or without ZrC particles, the survey spectra showed peaks corresponding to carbon (C), oxygen (O), and nitrogen (N). It is important to note that XPS does not detect hydrogen atoms.

Significant amounts of nitrogen (N) were detected in the coated RCF samples, with 15.6 atom% found in RCFs coated with 1% *w*/*v* PEI binders (a) and 0.8 atom% in 1% *w*/*v* PEI-coated RCFs with 30% *w*/*v* ZrC particles (a_2_). This indicates that the nitrogen content originates from the introduction of amino groups onto the reference RCFs through the PEI polymeric binder coating. In other words, the XPS analysis confirms the successful adsorption and precipitation of PEI on all RCFs in this study (a and a_2_). The presence of the PEI coating on the RCFs is also visually confirmed by the corresponding SEM micrographs. Furthermore, the increase in nitrogen content (atom%) for PEI-coated RCFs with ZrC particles, compared to the reference RCFs, was approximately 80%, further supporting the successful surface coating processes in this study.

The lower O/C ratio of sample a (0.23) compared to CV (0.55) is due to the nitrogen-rich composition of PEI, which reduces the relative oxygen content, as confirmed by the nitrogen content of sample a (15.6 at.%), absent in CV. In contrast, the higher O/C ratio of sample a_2_ (0.62) compared to a (0.23) suggests oxygen incorporation from surface oxides or adsorbed species. The gradual oxidation of ZrC, leading to the formation of ZrO_2_ and oxycarbides, likely contributed to the increased oxygen content detected by XPS.

The spectra confirm the presence of ZrC particles on the RCF surfaces, indicating successful attachment. Furthermore, for the RCFs functionalized with the PEI binder and ZrC particles, the XPS survey spectra clearly show the presence of ZrC, validating the successful incorporation of ZrC particles onto the fiber surfaces (Figure 5 a_2_).

### 4.2. Tensile Strength of RCFs

The tensile properties of both reference RCFs (CV) and coated fiber samples (a, a_1_, a_1.1_, a_2_, a_2.1_, b, b_1_, b_1.1_, bx, bx_1_, bx_1.1_) are summarized in Table 4. The increase in overall titer value (dtex) in the coated RCFs suggests the formation of a surface layer due to polymeric coatings and ZrC particle adsorption, contributing to the overall fiber mass. However, a relatively higher degree of variation in titer was observed in coated fibers (a_1_, a_1.1_, a_2_, a_2.1_, b_1_, b_1.1_, bx, bx_1_, bx_1.1_) compared to reference RCFs. This variation likely results from differences in ZrC particle attachment across fiber surfaces, excess aqueous solution retention between rollers during processing, and the non-uniform behavior of RCF bundles in polymeric coating mediums during stirring.

While the coatings contributed to increased fiber mass, they also influenced the mechanical performance. The overall tenacity values of coated RCFs exhibited a reduction, likely due to the adsorption of ZrC particles and interactions with the polymeric binders (PEI and poly-DADMAC). These coatings potentially induced a synergistic effect, restricting molecular mobility and reducing breaking strength. Additionally, elongation at break values of ZrC-coated samples (a_1_, a_1.1_, a_2_, a_2.1_, b_1_, b_1.1_, bx_1_, bx_1.1_) significantly decreased, which may be attributed to the aqueous coating medium interacting with ZrC particles. This interaction likely led to localized fiber structure loosening and disruption of hydrogen bonding within the cellulose matrix, thereby reducing flexibility.

Despite the observed decline in tenacity, the coated RCF samples (a, a_1_, a_1.1_, a_2_, a_2.1_, b, b_1_, b_1.1_, bx, bx_1_, bx_1.1_) remained within satisfactory limits for single-fiber tensile testability. This is an important consideration for potential downstream applications, including yarn spinning and fabric manufacturing. The results indicate that the applied coating protocol, incorporating optimized polymeric binder concentrations and ZrC particles, did not severely compromise the mechanical integrity or tensile performance of the fibers.

Moreover, these findings align with similar studies. For instance, Qi et al. [32] reported that MWCNT-coated RCFs, prepared via a scalable dip-coating process, exhibited no significant reduction in mechanical properties compared to uncoated regenerated cellulose fibers. While a slight increase in stiffness was noted, the CNT-coated fibers retained high flexibility and mechanical stability [32]. This comparison further supports the conclusion that the PEI- and polyDADMAC-coated RCFs, even with the inclusion of ZrC, maintain sufficient mechanical integrity for practical textile applications.

Furthermore, the highest level of reduction in coated RC fibers was observed in samples bx, bx_1_, and bx_1.1_. This could be attributed to the cross-linking effects of CaCl_2_ and CMC mixtures used in the pre-coating process, which may have contributed to a more rigid fiber structure due to the formation of stronger bonds.

In this context, our two distinctive polymeric binder solutions also contributed to preserving the mechanical properties of cellulose fibers, ensuring the applicability and reliability of the experimental setup for potential yarn spinning or nonwoven trials as one of the future targets of this study. Moreover, elongation at the break of all the coated RCFs decreased. This outcome could be attributed to the uniformity of the coating process’s effects on all these samples and serves as a key observation in this study.

Lastly, increasing the amount of polymeric binders, with or without ZrC particle attachment, may not be an effective strategy for enhancing the absorption and coating of polymeric-particle mixtures on regenerated cellulose fiber surfaces. Although maximizing ZrC particle deposition was a primary goal, experiments with higher concentrations of polyDADMAC and PEI (ranging from 2 to 10 wt.%) resulted in fiber structures that became progressively rigid, stiff, and brittle. In some cases, the coated fibers became inseparable, preventing titer measurement before tensile testing. For single-fiber testing, functionalized fibers must remain separable, flexible, and smooth enough for tensile evaluation while also being suitable for further processing in yarn spinning, nonwoven, or paper sheet applications. This limitation of our protocol, compared to studies on coating fabrics and yarns [26,27], directly impacts the amount of ZrC deposited on RCF surfaces and, consequently, the resulting heat retention properties. Notably, this serves as a key observation, confirming that our coating protocol optimizes the adsorption of polymeric binders and ZrC particles on RCF surfaces, making it the first study of its kind.

### 4.3. Heat Retention and Release Properties of the RCFs

**Figure 6:**
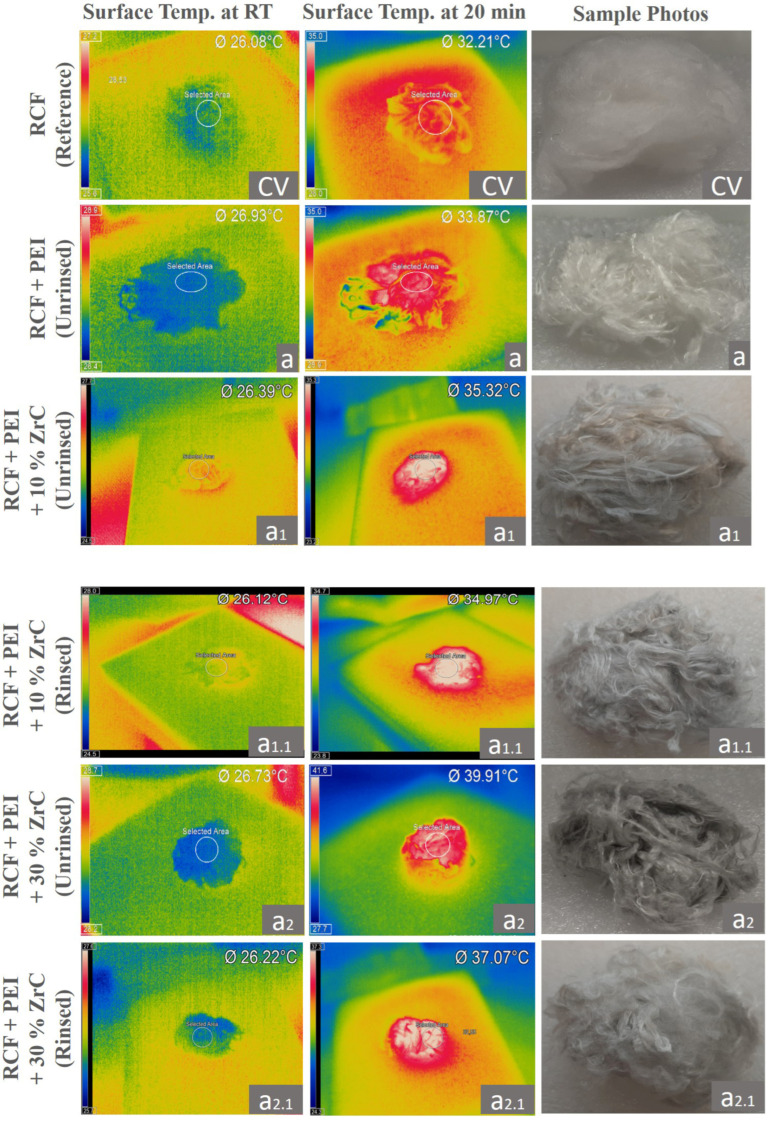
Infrared (IR) thermographic images (**left** and **middle**) and corresponding sample photos (**right**) of 1 g bundles of RCFs, including reference RCFs (CV), PEI-coated RCFs (a), and PEI-coated RCFs with 10 *w*/*v* % ZrC (a_1_: unrinsed, a_1.1_: rinsed) and 30 *w*/*v* % ZrC (a_2_: unrinsed, a_2.1_: rinsed). Images were taken at room temperature (RT, **left**) and after 20 min of heating (**middle**).

**Figure 7:**
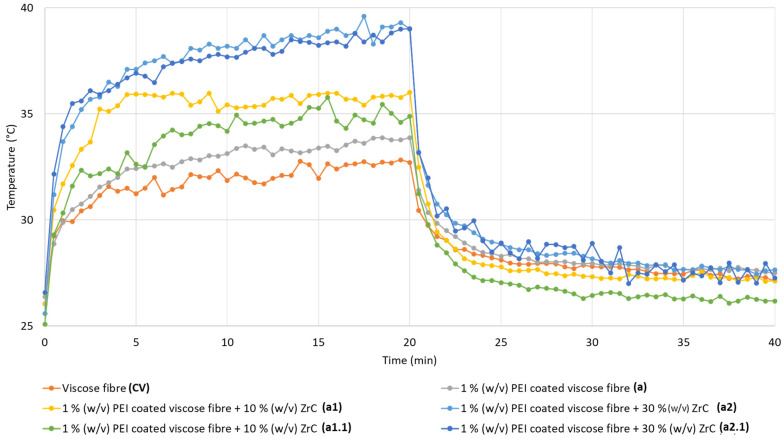
Dynamic curves of heat retention and release generated by IR radiation on RCF (CV, Viscose), PEI-coated RCFs (a), and PEI-coated RCFs with ZrC particles (a_1_, a_1.1_, a_2_, and a_2.1_), measured before and after IR irradiation using an IR lamp.

**Figure 8:**
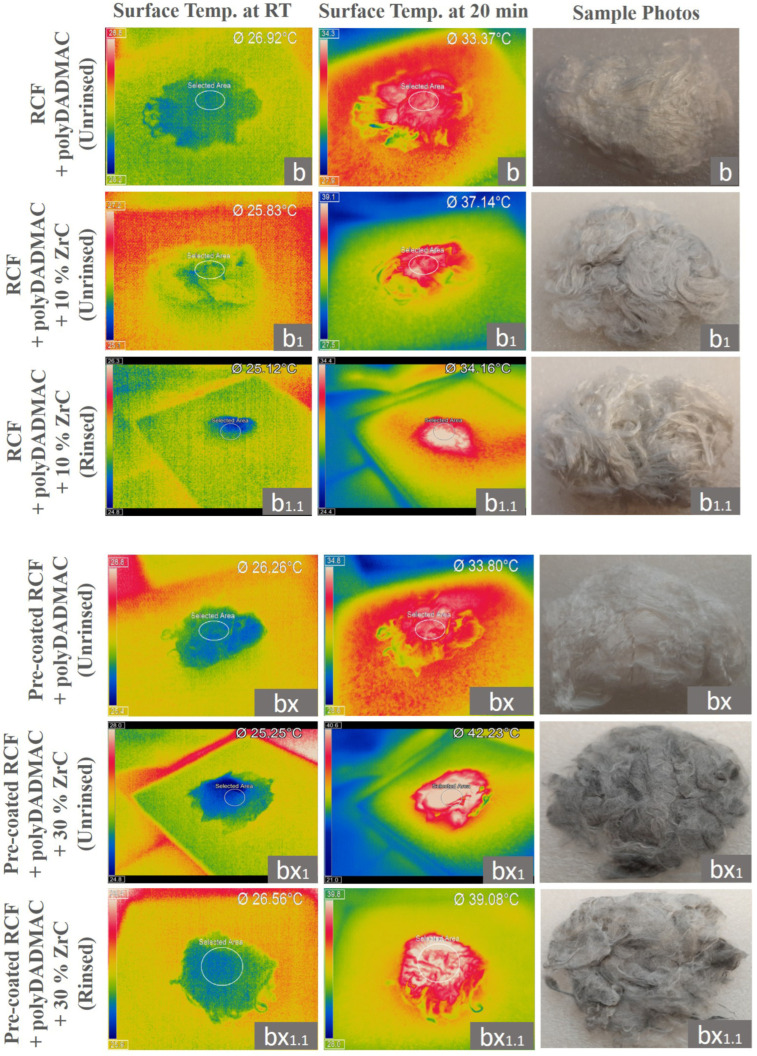
Infrared (IR) thermographic images (**left**,**middle**) and corresponding sample photos (**right**) of a 1 g bundle of RCFs (CV), polyDADMAC-coated RCFs (b), polyDADMAC-coated RCFs with 10 *w*/*v* % ZrC (b_1_: unrinsed, b_1.1_: rinsed), pre-coated RCFs with a CaCl_2_ + CMC mixture (bx), and the pre-coated RCFs (bx) with additional poly-DADMAC-coated fibers and 30 *w*/*v* % ZrC particles (bx_1_: unrinsed, bx_1.1_: rinsed). Images were taken at room temperature (RT, **left**) and after 20 min of heating (**middle**).

**Figure 9:**
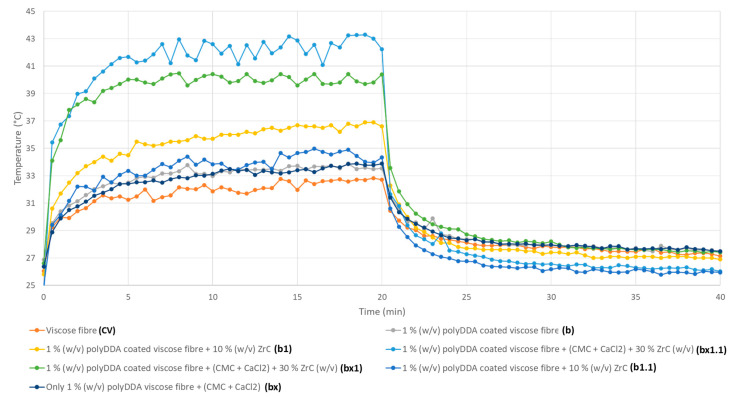
Dynamic curves of IR heat retention and release of RCFs (CV, Viscose) and coated RCFs with polyDADMAC (b), polyDADMAC-coated RCFs with ZrC (b_1_, b_1.1_), and pre-coated RCFs with a CaCl_2_ + CMC mixture followed by polyDADMAC coating (bx), with and without ZrC (bx_1_, bx_1.1_ and bx).

The heat retention and release characteristics of the fiber samples, with and without polyDADMAC and PEI binders and ZrC particles, were evaluated using an infrared (IR) light source and continuously monitored with an IR camera (Figure 6, Figure 7, Figure 8 and Figure 9). All RCF samples were exposed to identical heat radiation under controlled laboratory conditions. However, variations in their temperature increase were observed, influenced by the presence of polymeric binders and the amount of coated ZrC particles.

The heat emission from the RCFs of this study refers to the thermal energy radiated after exposure to an infrared (IR) light source [36]. Figure 6 and Figure 7 display IR thermographic images and the heat-emission diagram, illustrating surface temperature variations in ZrC-coated fibers and reference RCF specimens, with and without PEI binders, under IR lamp radiation as a function of irradiation time.

Figure 6 illustrates the surface temperature increase in fiber bundles over time under IR radiation from a light source positioned 50 cm above the specimens, as shown in Figure 2. The ZrC-deposited RCFs (a_1_: 26.7 °C to 39.9 °C, a_1.1_: 26.2 °C to 37.1 °C, a_2_: 26.4 °C to 35.3 °C, a_2.1_: 26.1 °C to 34.5 °C) exhibited higher temperatures than the reference RCFs (CV) and the PEI-coated fibers (a: 26.0 °C to 32.2 °C) under identical IR irradiation conditions. This suggests that the greater temperature increase is primarily due to the strong IR light absorption capacity of the higher amount of coated ZrC particles.

Furthermore, the RCF samples with a higher ZrC content (a_1_: 10% *w*/*v* to a_2_: 30% *w*/*v*) displayed a more pronounced non-linear temperature increase (from 37.3 °C to 39.9 °C) than the reference RCFs (CV: 32.2 °C) and the PEI-coated fibers without ZrC (a: 33.8 °C) over 20 min of IR exposure. The final temperatures of the ZrC-coated fibers were directly proportional to the amount of ZrC bound with PEI, demonstrating an effective photo-thermal conversion process. A similar correlation was reported by Xu et al. [5], where the surface temperature of ZrC-coated PES fabric increased from 38 °C (untreated) to 41.2 °C after their ZrC incorporation.

Although the results from Kim and Kim [29] may not be directly comparable with our study due to the differences between test setups and conditions, since they used PET fabric and a thermometer for thermal radiation measurements instead of an IR camera, the effect of ZrC on heat retention can still be assessed. In their study, after 10 min of IR exposure, the surface temperature of ZrC-coated PET fabric increased non-linearly from 34 °C to 38 °C [29], which is in line with our findings for our ZrC-deposited RCFs (a_1_: 26.7 °C to 39.9 °C, a_1.1_: 26.2 °C to 37.1 °C, a_2_: 26.4 °C to 35.3 °C, a_2.1_: 26.1 °C to 34.5 °C). This validates our method and further reinforces the effectiveness of ZrC particles in improving heat retention for RCF samples.

During the initial 5–10 min of irradiation, the surface temperatures of the ZrC-coated RCFs increased more rapidly than those of the reference RCFs, demonstrating the immediate impact of the ZrC coating (Figure 6 and Figure 7). However, as the heating period extended to 20 min, the temperature increase plateaued, indicating a stabilization in heat absorption and retention. This highlights the thermoregulation capability of the ZrC-coated RCFs in this study.

Notably, the surface temperature of our ZrC-coated RCF samples stabilized within the range of 37–39 °C, an optimal temperature for human clothing that ensures thermo-physical comfort by aligning with the natural body temperature. In contrast, if the heat emission of the ZrC-coated RCFs had exceeded 39–40 °C under IR exposure, it could lead to discomfort for potential wearers, making it unsuitable for the intended application. This aspect is crucial, as the study aims to develop coated RCFs that can be further processed into yarns and fabrics for functional clothing textiles.

Ultimately, the stabilization of the maximum temperature of the ZrC-coated RCFs with PEI binders (a_1.1_: 26.2 °C to 37.1 °C) within the ideal range after 20 min of IR exposure highlights the practical significance of this study. It confirms that the developed RCF coating effectively supports thermoregulating textiles by maintaining a balance between heat retention and wearer comfort.

Furthermore, the durability of the heat-generating effect was assessed by monitoring the surface temperature of the fiber samples after the IR radiation source was switched off. Once the IR light source was turned off after 20 min of heating, all samples exhibited a rapid temperature drop to approximately 25 °C within the next 20 min. This rapid exothermic response highlights the superior heat-releasing capability of PEI + ZrC-coated RCFs compared to uncoated RCFs.

The heat-releasing temperatures, defined as the final temperature drop through heat dissipation after IR irradiation, were higher for PEI-coated, ZrC-embedded RCF samples (a_1_, a_1.1_, a_2_, and a_2.2_) than for the reference RCFs (CV). This effect is likely attributed to the release of absorbed IR radiation retained by the ZrC particles coated on the RCF bundle [29,31].

Notably, the surface temperature of 30% (*w*/*v*) ZrC-deposited RCF samples decreased to a level comparable to that of 10% (*w*/*v*) ZrC-coated fibers within 10 s after the IR light was switched off, as shown in Figure 6. After 240 s, the temperatures of both 10% and 30% (*w*/*v*) ZrC-coated RCF samples had dropped to nearly the same level as the reference RCF (CV). This indicates that ZrC-functionalized RCFs possess a strong capacity for rapid exothermicity, efficiently releasing stored heat within a short time. A similar phenomenon was reported by Li et al. in their study on the heat-generating properties of ZrC-doped RCFs in nonwoven form [7].

It was also observed that RCFs modified with PEI binders exhibited only a slight increase, approximately 1 °C, in released temperature compared to the reference RCF after 20 min of IR light exposure. This indicates that the PEI polymeric solution has a negligible impact on the heat release and storage properties of ZrC-embedded RCF.

In addition, rinsed fiber samples (a_1.1_, a_2.2_), which were coated with both ZrC and PEI, exhibited slightly lower heat release temperatures (35–37 °C) compared to the unrinsed samples (a_1_, a_2_). However, these values still represent a significant improvement over the heat release of uncoated RCFs (32.2 °C). Notably, sample a_1.1_ reached a temperature of 37.07 °C, which is particularly relevant as it closely aligns with the natural body temperature of a healthy human [37].

Figure 8 and Figure 9 illustrate the IR thermographic images and IR light heat-emission diagram of the surface temperature of ZrC-coated RCFs and reference RCFs (CV) fiber specimens with polyDADMAC binders (b, b_1_, b_1.1_, bx, bx_1_, and bx_1.1_), both with (b_1_, b_1.1_, bx_1_, and bx_1.1_) and without ZrC particles (b and bx) under IR lamp radiation as a function of irradiation time. Similar the results obtained for ZrC-coated samples with PEI binders (a_1_, a_1.1_, a_2_, and a_2.2_) are shown in Figure 6. Figure 8 demonstrates the temperature rise on the ZrC-coated RCF bundle surfaces with polyDADMAC binder (b_1_, b_1.1_, bx, bx_1_, and bx_1.1_) over time as IR light was emitted from a source positioned 50 cm above the specimens (see Figure 2).

The ZrC-deposited RCFs (b_1_, b_1.1_, bx_1_, and bx_1.1_), both rinsed (b_1.1_ and bx_1.1_) and unrinsed (b_1_ and bx_1_), exhibited significantly higher temperature increases compared to the reference RCFs and the polyDADMAC-coated RCFs (b), confirming the strong IR light absorption capacity of ZrC particles. Specifically, sample b_1_ exhibited a temperature rise from 25.8 °C to 37.1 °C, while b_1.1_ increased from 25.1 °C to 34.1 °C. Similarly, the bx sample showed a rise from 26.2 °C to 33.8 °C, whereas the bx_1_ sample reached the highest temperature, increasing from 25.2 °C to 42.2 °C. Sample bx_1.1_ also demonstrated a notable increase from 26.5 °C to 39.1 °C. In contrast, the polyDADMAC-coated RCF sample (b) exhibited a lower temperature rise, from 26.9 °C to 33.8 °C.

These findings further indicate that the enhanced heat retention and emission properties of the RCF samples are primarily attributed to the IR light absorption capacity of ZrC particles.

The images in Figure 7 illustrate the heat absorption and retention properties of these RCF samples under IR irradiation. Similar to the PEI-coated samples with 10% and 30% (*w*/*v*) ZrC (a_1_, a_1.1_ and a_2_, a_2.1_), the polyDADMAC-coated RCFs with coated ZrC also exhibited a non-linear increase in surface temperature during 20 min of IR lamp exposure. Specifically, RCF samples coated with 10% (*w*/*v*) ZrC (b_1_) showed a temperature rise from 25.8 °C to 37.1 °C, while those with 30% (*w*/*v*) ZrC (bx_1_) reached 42.2 °C under the same conditions. Comparable findings of those temperature increases were previously reported for ZrC-embedded PET woven fabric samples and PET-Nylon blended yarns under 10 min of IR-light exposure, as demonstrated in studies by Kim and Kim [29] and Kim et al. [31], respectively.

The ultimate surface temperatures of polyDADMAC-coated RCF samples correlated with their ZrC content, aligning with findings by Li et al. [7], who developed photoinduced heat-generating viscose fibers with 1–4% ZrC, and Wang et al. [38], who fabricated polyurethane-bonded cotton yarns with 3–6% ZrC for photothermal conversion applications. Additionally, RCFs pre-coated with a CMC and CaCl_2_ mixture, followed by polyDADMAC coating with 30% (*w*/*v*) ZrC (bx_1_), reached a peak temperature of 42.2 °C, while the rinsed counterpart (bx_1.1_) achieved 39.8 °C.

In contrast, RCFs coated with polyDADMAC binders (b) and pre-coated with a CaCl_2_ and CMC mixture before polyDADMAC coating (bx) exhibited minimal heat retention, with a temperature increase in just 1–1.5 °C, compared to the reference RCFs (CV). This indicates that the polyDADMAC binder alone does not significantly enhance the heat-storage properties of ZrC-coated RCF samples. However, rinsed RCF samples (b_1.1_, bx_1.1_) coated with both ZrC and polyDADMAC reached slightly lower surface temperatures (37.1 °C and 39.8 °C, respectively), compared to their unrinsed counterparts (b_1_: 42.2 °C, bx_1_: 39.8 °C). Despite this reduction in surface temperature of the RCF samples (b_1,_ b_1.1_, bx_1,_ bx_1.1_), attributed to typical effects of the rinsing process removing unbound polyDADMAC and ZrC, these values (bx_1.1_: 39.8 °C and b_1.1_: 34.1 °C) still represent a notable improvement over the reference RCFs, which reached only 32.2 °C under 20 min of IR light exposure.

Finally, when the IR light source was turned off after 20 min of heating, the surface temperature of all polyDADMAC-coated RCF samples, both with and without ZrC particles (b_1_, b_1.1_, bx_1_, and bx_1.1_), rapidly dropped to approximately 25 °C within the next 20 min. This cooling behavior was similar to that observed in PEI-coated RCFs (a, a_1_, a_1.1_, a_2_, and a_2.1_), with and without ZrC particles, in our study.

Among all fiber samples in this study, the highest surface temperatures were recorded for 1.0% (*w*/*v*) polyDADMAC-coated RCFs pre-coated with a CaCl_2_ + CMC mixture and coated with 30% (*w*/*v*) ZrC, reaching 42.2 °C (unrinsed bx_1_) and 39.1 °C (rinsed bx_1.1_). Similarly, 1.0% (*w*/*v*) PEI-coated RCFs with 30% (*w*/*v*) ZrC reached 39.9 °C (unrinsed a_1_) and 37.1 °C (rinsed a_1.1_). These findings indicate that while PEI and polyDADMAC polymeric binders (with or without CaCl_2_ + CMC pre-coating on the RCF surfaces) had a negligible effect on the heat generation performance of coated RCF samples, the presence of CaCl_2_ + CMC notably enhanced ZrC coating and thermal performance.

The key difference between PEI-coated (Figure 6 and Figure 7) and polyDADMAC-coated (Figure 8 and Figure 9) RCF samples, especially with ZrC particles, stems from the pre-coating effect of CaCl_2_ and CMC mixtures, which acted as a crosslinker with polyDADMAC, enhancing ZrC particle adsorption. As a result, samples bx, bx_1_, and bx_1.1_ exhibited a higher ZrC content, leading to improved heat retention properties, particularly for bx_1_ (unrinsed) and bx_1.1_ (rinsed), compared to a_2_ and a_2.1_ coated with PEI. In summary, the pre-coating process enhanced both ZrC adsorption capacity and rinsing performance, as evidenced by the higher heat retention of bx_1.1_ compared to a_2.1_.

Both PEI and polyDADMAC are well-known polyelectrolyte binders, commonly used as flocculating agents due to their high affinity for inorganic particle absorption and retention [39,40]. In this study, these binders were employed with a similar goal, maximizing ZrC particle adsorption on RCFs to enhance their heat-generating properties. Notably, pre-coating with CaCl_2_ + CMC mixtures for cross-linking polyDADMAC on regenerated cellulose fibers (RCFs) has been outlined in a patent for the surface modification of cellulose fibers using CaCl_2_ + CMC mixtures [41]. However, prior studies have not explored their application for surface coating of RCFs with polyDADMAC, making our research the first to demonstrate the synergistic effects of CaCl_2_ + CMC and polyDADMAC. This novel approach of pre-coating RCFs with a CaCl_2_ and CMC mixture offers significant potential for effectively embedding and retaining ZrC particles on fiber surfaces through subsequent polyDADMAC coating. Additionally, it can be applied to various textile forms, including (non)wovens, fabrics, and yarns. Most importantly, our study is the first to explore and report the use of this technique for photo-induced heat generation, retention, and release.

## 5. Conclusions

This study presented a facile coating procedure for introducing ZrC particles onto RCF surfaces using two distinct polymer binders: PEI and polyDADMAC. The objective was to enhance heat retention and release properties of the RCFs, which is the first study of its kind.

- SEM micrographs confirmed the successful coating of ZrC particles on RCFs;
- The coatings exhibited notable thermal properties:
  ○ The surface temperatures of 30% (*w*/*v*) ZrC-embedded fibers coated with PEI binders were 37.1 °C and 39.8 °C, significantly higher than the reference RCFs (32.2 °C);
  ○ The addition of CMC and CaCl_2_ improved the coating efficiency of polyDADMAC binders;
  ○ The highest temperature increase was observed in infrared (IR) heat emission diagrams for polyDADMAC-coated fibers with a CaCl_2_ + CMC mixture and ZrC coating, both rinsed and unrinsed (b_x1_: 42.23 °C; b_x1.1_: 39.08 °C);
  ○ These findings highlight the suitability of the developed clothing textiles for sustainable applications in non-extreme thermal conditions, ensuring thermo-physiological comfort by maintaining body temperature within a tolerable thermal range (36.5–37.5 °C).
- A moderate decrease in tensile strength (tenacity and elongation at break) was observed compared to reference RCFs. However, the following was noted:
  ○ This reduction did not affect fiber flexibility, making them suitable for further yarn spinning and fabric production.
- XPS spectra provided qualitative confirmation of PEI attachment onto RCFs, with and without ZrC particle coating. These findings aligned with the SEM micrograph observations;
- The ZrC-coated RCF samples in this study stabilized at an optimal temperature range of 37–39 °C, ensuring thermo-physical comfort by aligning with natural body temperature. If the heat emission exceeded 39–40 °C under IR exposure, it could compromise wearer comfort, making it unsuitable for our intended application. These findings confirm that our coating approach effectively enhances heat retention while maintaining comfort, supporting the study’s aim of developing RCF-based materials for functional clothing textiles.

### Future Perspectives of This Study

Once larger quantities of the developed RC fibers become available, further studies will be conducted to comprehensively assess their practicality, comfort, and suitability for use in clothing. Given their exceptional properties, these materials have the potential to significantly advance functional textiles. Additionally, various tests, including far-infrared emissivity analysis, will be conducted to explore their potential biomedical applications.

## Figures and Tables

**Table 1:** Key data and test conditions on the photothermal conversion-based heat retention and release properties of various ZrC-embedded (non-)cellulosic materials, fabricated using different methods, compared to the results of this study, which focuses on functional clothing textiles for non-extreme thermal conditions.

Type of Textiles Used/Fabricated with Incorporated ZrC Particles	Experimental Conditions for the IR Light Heat Emission Setup		Data for Photothermal Conversion-Based Heat Retention (Δ = Heat Increase Between Reference and Max temp., °C) (Rate = +%)	Ref.
	IR Lamp	IR Camera/Sensor		
Surface-coated RCF with PEI	IR lamp (Philips, 100 W 230 V R95)	IR camera (Optris PI400)	Reference RC fiber: 32.21 °C ZrC-coated RC fiber: 37.07 °C (Δ = Heat increase, 4.86 °C) (Rate = +15.09%)	This study
	Distance: 50 cm (from the sample)	Distance: 20 cm (from the sample)		
	IR light test: Light-on (20 min) Light-off (20 min)	Specimens tested at 20 ± 2 °C and RH of 50 ± 2%.		
Surface-coated RCF with polyDADMAC (pre-coated with CaCl_2_ + CMC)	IR lamp (Philips, 100 W 230 V R95)	IR camera (Optris PI400)	Reference RC fiber: 32.21 °C ZrC-coated RC fiber: 39.08 °C (Δ = Heat increase: 6.87 °C) (Rate = +21.34%)	This study
	Distance: 50 cm (from the sample)	Distance: 20 cm (from the sample)		
	IR light test: Light-on (20 min) Light-off (20 min)	Specimens tested at 20 ± 2 °C and RH of 50 ± 2%.		
Spunlace nonwoven with mélange fibers containing Heat Wave^TM^ (*)	Reflector lamp (500 W)	N/A: Camera/Sensor	Reference RC fiber: ≈33 °C ZrC-coated RC fiber: ≈38 °C (Δ = Heat increase: ≈5 °C) (Rate = +15.15%)	[30]
	Distance: 50 cm (from the sample)	Nonwovens tested at 20 ± 2 °C and RH: N/A		
	IR light test: Light-on (20 min) Light-off (15 min)	Closest material, and test setup to our study.		
Knitted PES fabric with PES-textured filament Solar α™ (**)	REF lamp (PRF-500 W), central luminosity: 6000 cd)	Thermosensor on an insulator.	Reference PES knit fabric: ~23 °C ZrC-PES fabric: 26 °C (Δ = Heat increase, 3 °C) (Rate = +13.04%)	[28]
	Distance: 80 cm (from the sample)	N/A: Specimen size N/A: Test conditions		
Woven PET fabric with core-spun bicomponent PET yarn	Heat emission bulb (220 V/500 W/3200 K)	Thermometer N/A: Heat-insulator	Reference PET woven fabric: 34.8 °C ZrC-embedded PET fabric: 38 °C (Δ = Heat increase, 3.2 °C) (Rate = +9.20%)	[29]
	Distance: 50 cm (from the sample)	Specimens prepared a 20 ± 2 °C and RH of 64 ± 4%.		
	IR light test: Light-on (10 min) Light-off (20 min)			

(*) Heat Wave™ is a heat-generating viscose (RC) fiber, pre-fabricated by Daiwabo Rayon Co., Ltd. (Osaka, Japan), and tested by the Kaken Test Center in Tokyo, Japan. The test parameters and results were obtained from the datasheet available on the company’s website. The mélange fibers consist of cotton/Heat Wave™ (80/20) and cotton/reference viscose (80/20). (**) Solar α™ is a heat-generating PES-textured filament containing 2% ZrC, developed by Unitika Ltd. in Osaka, Japan.

**Table 2:** Abbreviations and descriptions of the reference RCF (CV) used and the (pre-)coated RCF samples fabricated in the experiments.

Sample Abbreviations	Sample Descriptions
RCF, (CV)	Reference RCF without any (pre-)coating
(a)	Coated RCFs with 1% (*w*/*v*) PEI (unrinsed)
(a_1_)	Coated RCFs with 1% (*w*/*v*) PEI with 10% (*w*/*v*) ZrC (unrinsed)
(a_1.1_)	Coated RCFs with 1% (*w*/*v*) PEI with 10% (*w*/*v*) ZrC (rinsed)
(a_2_)	Coated RCFs with 1% (*w*/*v*) PEI with 30% (*w*/*v*) ZrC (unrinsed)
(a_2.1_)	Coated RCFs with 1% (*w*/*v*) PEI with 30% (*w*/*v*) ZrC (rinsed)
(b)	Coated RCFs with 1% (*w*/*v*) polyDADMAC (unrinsed)
(b_1_)	Coated RCFs with 1% (*w*/*v*) polyDADMAC with 10% (*w*/*v*) ZrC (unrinsed)
(b_1.1_)	Coated RCFs with 1% (*w*/*v*) polyDADMAC with 10% (*w*/*v*) ZrC (rinsed)
(bx)	Pre-coated RCFs with (0.5 M CaCl_2_ + 5% *w*/*v* CMC) mixture and coated with 1% (*w*/*v*) polyDADMAC (unrinsed)
(bx_1_)	Pre-coated RCFs with (0.5 M CaCl_2_ + 5% *w*/*v* CMC) mixture and coated with 1% (*w*/*v*) polyDADMAC with 30% (*w*/*v*) ZrC particles (unrinsed)
(bx_1.1_)	Pre-coated RCFs with (0.5 M CaCl_2_ + 5% *w*/*v* CMC) mixture and coated with 1% (*w*/*v*) polyDADMAC (unrinsed) with 30% (*w*/*v*) ZrC particles (rinsed)

**Table 3:** The elemental surface compositions of RCF and PEI-coated RCFs with and without ZrC.

RCF Samples	Surface Compositions (at.%)			
	Oxygen (O)	Carbon (C)	O/C Ratio	Nitrogen (N)
(CV) Reference RCF	34.9	63.1	0.55	-
(a) Coated RCFs with 1% (*w*/*v*) PEI	15.6	68.5	0.23	15.6
(a_2_) Coated RCFs with 1% (*w*/*v*) PEI + 30% (*w*/*v*) ZrC	37.4	60.7	0.62	0.8

**Table 4:** Tensile properties of the RCFs and all the coated fiber samples.

Specifications of the Tested Samples	Titer (dtex)	Tenacity (cN/tex)	Elongation (%)
Regenerated cellulose fiber (RCF) (CV)	1.41 ± 0.2	23.7 ± 2.3	20.4 ± 2.6
Coated RCF with 1% (*w*/*v*) PEI (unrinsed) (a)	1.44 ± 0.2	21.9 ± 1.7	19.2 ± 2.1
Coated RCF with 1% (*w*/*v*) PEI + 10% (*w*/*v*) ZrC (unrinsed) (a_1_)	1.45 ± 0.3	21.3 ± 3.1	19.1 ± 1.3
Coated RCF with 1% (*w*/*v*) PEI + 10% (*w*/*v*) ZrC (rinsed) (a_1.1_)	1.43 ± 0.4	23.4 ± 0.9	15.4 ± 3.3
Coated RCF with 1% (*w*/*v*) PEI + 30% (*w*/*v*) ZrC (unrinsed) (a_2_)	1.47 ± 0.6	20.0 + 5.2	12.9 ± 3.3
Coated RCF with 1% (*w*/*v*) PEI + 30% (*w*/*v*) ZrC (rinsed) (a_2.1_)	1.44 ± 0.3	22.3 + 3.4	14.1 ± 2.4
Coated RCF with 1% (*w*/*v*) polyDADMAC (unrinsed) (b)	1.35 ± 0.1	20.3 ± 1.8	15.1 ± 2.3
Coated RCF with 1% (*w*/*v*) polyDADMAC + 10% (*w*/*v*) ZrC (rinsed) (b_1_)	1.50 ± 0.4	21.6 ± 1.4	15.8 ± 2.5
Coated RCF with 1% (*w*/*v*) polyDADMAC + 10% (*w*/*v*) ZrC (rinsed) (b_1.1_)	1.52 ± 0.3	19.9 ± 2.3	17.9 ± 2.3
Pre-coated RCFs with CMC + CaCl_2_ mixture, then coated with 1% (*w*/*v*) polyDADMAC (unrinsed) (bx)	1.51 ± 0.4	19.7 ± 1.5	16.7 ± 1.7
Pre-coated RCFs with CMC + CaCl_2_ mixture, then coated with 1% (*w*/*v*) polyDADMAC and 30% (*w*/*v*) ZrC (unrinsed) (bx_1_)	1.45 ± 0.1	17.8 ± 6.8	15.8 ± 6.0
Pre-coated RCFs with CMC + CaCl_2_ mixture, then coated with 1% (*w*/*v*) polyDADMAC and 30% (*w*/*v*) ZrC (rinsed) (bx_1.1_)	1.50 ± 0.1	19.1 ± 2.3	14.9 ± 2.8

## Data Availability

The original contributions presented in this study are included in the article material. Further inquiries can be directed to the corresponding author.
